# Relationships between Values of Antibodies to Several Connective Tissue Disease Autoantigens and Pulmonary Function in a Japanese General Population: The Takahata Study

**DOI:** 10.1371/journal.pone.0081678

**Published:** 2013-12-03

**Authors:** Hiroshi Nakano, Yoko Shibata, Sumito Inoue, Akira Igarashi, Keiko Yamauchi, Shuichi Abe, Masamichi Sato, Yasuko Aida, Keiko Nunomiya, Tomomi Kimura, Takako Nemoto, Tetsu Watanabe, Tsuneo Konta, Yoshiyuki Ueno, Takeo Kato, Takamasa Kayama, Isao Kubota

**Affiliations:** 1 Department of Cardiology, Pulmonology and Nephrology, School of Medicine, Yamagata University, Yamagata City, Yamagata, Japan; 2 Global Center of Excellence Program Study Group, School of Medicine, Yamagata University, Yamagata City, Yamagata, Japan; Keio University School of Medicine, Japan

## Abstract

**Background:**

Accumulating evidence suggests the involvement of an autoimmune mechanism in the pathogenesis of respiratory dysfunction. The aim of this study was to investigate the relationship between pulmonary function and serum antibodies to several connective tissue disease autoantigens (ACTDA) levels, which has not been investigated in a general population.

**Methods:**

Blood sampling and spirometry were performed for subjects (n = 3,257) aged ≥40 years who participated in a community-based annual health check in Takahata, Japan, from 2004 to 2006. ACTDA was measured by enzyme immunoassay, and subjects with ACTDA values ≥20 were defined as positive.

**Results:**

In males, there were significant inverse relationships between logarithmically transformed ACTDA values and spirometric parameters, including % predicted values for forced expiratory volume in 1 s (FEV_1_) and maximal midexpiratory flow (MMF) as well as FEV_1_/forced vital capacity (FVC). Multiple linear regression analysis revealed that except for the relationship between ACTDA and FEV_1_/FVC, these relationships were still significant after adjustment for Brinkman index (a measure of inhaled cigarette consumption). The prevalence of positive ACTDA was greater in male never-smokers with mixed ventilation disorders and relatively severe airflow obstruction (% predicted FEV_1_ below the median value).

**Conclusions:**

Autoimmunity may be involved in the mechanism of impaired pulmonary function in the general population.

## Introduction

Inhalation of cigarette smoke causes respiratory inflammation even in healthy smokers, and long-term smoking causes various respiratory diseases. Of these diseases, chronic obstructive pulmonary disease (COPD) has had the most impact on public health worldwide [1]. In patients with COPD, forced expiratory volume in 1 s (FEV_1_) is progressively reduced with the development of this disease. Imbalances of proteases/antiproteases and oxidants/antioxidants were thought to be major mechanisms underlying the development of COPD, but accumulating evidence has recently suggested that systemic inflammation due to the leakage of mediators from sites of local inflammation plays an important role in its pathogenesis [2].

In addition, autoimmunity is associated with the pathogenesis of pulmonary diseases such as COPD [3]. For instance, lymphoid follicles containing B cells accumulate in the airways of patients with COPD [4], and Nunez et al. reported that the anti-tissue antibodies such as mitochondrial, liver-kidney microsomal smooth muscle, and parietal gastric cell antibodies are associated with the degree of airflow limitation in COPD patients [5]. Therefore, autoantibody-induced airway damage may contribute to the pathogenesis of the disease. In particular, the involvement of autoimmune mechanisms is suggested by the persistence of airway inflammation after cessation of cigarette smoking [6] as well as by the pathogenesis of COPD in non-smoking populations [7].

The prevalence of antinuclear antibody (ANA) in the general population is 13.8% in the United States and 26% in Japan [8,9]. We previously reported spirometric values for a healthy Japanese population ≥40 years of age who participated in an annual health check [10], noting that the prevalence of microalbuminuria, a marker of vascular endothelial damage, was significantly greater in the group measuring positive for antibodies to several connective tissue disease autoantigens (ACTDA), as measured using an enzyme immunoassay (EIA) method, than in the ACTDA-negative group [11]. 

It has been reported that there is no relationship between cigarette smoking and ANA positivity [7,9]. In contrast, the involvement of cigarette smoking in the development of autoimmune diseases has been suggested [12]. If this is true, cigarette smoking may cause airway inflammation by dual pathways: direct impairment by the toxicity of smoke which triggers the inflammatory response, and indirect impairment by the production of autoantibodies which triggers the inflammation in the airway epithelial and/or endothelial cells. In COPD, pulmonary endothelial dysfunction is an important feature of its pathogenesis [13,14].

To date, the association of antinuclear antibodies with spirometric values in the general population has not been elucidated. In this study, we cross-sectionally and longitudinally investigated the relationship between ACTDA values and spirometric parameters in a general Japanese population.

## Methods

### Study population

This study was part of the Molecular Epidemiological Study utilizing the Regional Characteristics of 21^st^ Century Centers of Excellence (COE) Program and the Global COE Program in Japan [10,15-18]. The study was approved by the ethics committee of Yamagata University School of Medicine, and all participants gave written informed consent.

This study was based on an annual community health check, in which all residents of Takahata (in northern Japan) ≥40 years of age were invited to participate. From 2004 through 2006 (visit 1), 1,579 men and 1,941 women (a total of 3,520 subjects) were enrolled in the study and underwent initial spirometry. Two hundred sixty-three subjects were excluded from the analysis because spirometry data did not meet the criteria described below; data for 3,257 subjects (1,502 males and 1,755 females) were entered into the final statistical analysis. Subjects used a self-report questionnaire to document their medical histories, smoking habits, current use of medications, and clinical symptoms. One hundred forty-seven of the 542 male current smokers at visit 1 underwent subsequent follow-up spirometry in 2009 (visit 2) [19].

### Measurements

Fasting blood samples were obtained from an antecubital vein and immediately transferred to chilled tubes. The presence of ACTDA in serum was determined using an enzyme immunoassay (EIA) method (Medical & Biological Laboratories Co. Ltd., Nagoya, Japan). We used the MESACUP ANA EIA kit, that utilized a mixture of purified recombinant or natural antigens, including single- and double-stranded DNA, RNP, Sm, SS-A/Ro, SS-B/La, centromere, topoisomerase I, and Jo-1 antigens. Because EIA detects antibodies to a limited number of autoantigens, the value obtained using this kit is not identical to an ANA titer measured by indirect immunofluorescence assay (IIF); thus, we applied the term *ACTDA* rather than ANA in this manuscript. An ACTDA index ≥20.0 was defined as positive, in accordance with the manufacturer’s reference materials [11].

Spirometric parameters of forced vital capacity (FVC) and FEV_1_ were measured using standard techniques, with subjects performing FVC maneuvers on a CHESTAC-25 part II EX instrument (Chest Corp., Tokyo, Japan) in accordance with the guidelines of the Japanese Respiratory Society (JRS) [20]. Bronchodilators were not administered before spirometry. The highest value of at least 3 FVC maneuvers performed by each subject was used for the analysis. The results were assessed by 2 pulmonary physicians, who visually inspected the flow-volume curves and excluded subjects with data that were inadequate, as defined by JRS criteria [20]. The rate of decline in spirometric measures was calculated as follows: [(% predicted value at visit 2 – % predicted value at visit 1)/% predicted value at visit 1] × 100/time between observations (years) [21]. The main spirometric reasons for exclusion from the study were sustained cough and unacceptable delay of peak flow during expiration due to insufficient effort.

### Statistical analysis

For continuous variables, data are presented as the mean (standard deviation [SD]). Student’s t-test for parametric data and the Mann-Whitney U test for non-parametric data were used to analyze the differences between 2 groups. Analysis of variance followed by Tukey’s test was performed for multivariate comparisons. Univariate regression analysis was used to examine the association between log ACTDA levels and each spirometric measure considered in this study. Multiple linear regression analysis was then performed to determine whether log ACTDA levels contributed to each of these spirometric measures after adjustment for all other variables included in the model. Differences in proportions were evaluated using the chi-square test. Statistical significance was inferred for two-sided *P* values <0.05. All statistical analyses were performed using JMP version 8 software (SAS Institute Inc., Cary, NC, USA).

## Results

### Comparison of characteristics of ACTDA-positive and ACTDA-negative subjects

Of the subjects who underwent spirometry on visit 1, 191 males and 372 females were positive for ACTDA. The distribution of ACTDA measurements was skewed towards higher values (25% quartile, 7.2; median, 11.1; 75% quartile, 17.2). Therefore, ACTDA values were logarithmically transformed before analysis. Logarithmically-transformed ACTDA values were significantly associated with age in both males (coefficient, 5.75; *P* < 0.0001) and females (coefficient, 3.06; *P* = 0.0007). In addition, they were associated with Brinkman index (number of cigarettes per day × years of smoking) in males (coefficient, 154.05; *P* = 0.01) but not in females (coefficient, –5.304; *P* = 0.51). Characteristics were compared according to the level of ACTDA. An ACTDA index ≥20.0 was defined as positive, in accordance the manufacturer’s reference materials [11]. In addition, an ACTDA index of 40.0 is nearly the value of the mean + (2 × SD). Thus, we stratified the population using these cutoffs (Tables 1, 2, 3, 4, 5 and 6). A higher level of ACTDA was associated with higher mean age (Tables 1 and 4). Brinkman index and proportion of former or current smokers were not significantly different among 3 groups (Tables 1 and 4). However, higher ACTDA index was associated with the lower FVC% predicted, FEV_1_% predicted, FEV_1_/FVC, and maximal midexpiratory flow (MMF)% predicted in male subjects, while these associations were not observed in women (Tables 1 and 4). In subjects aged <65 years, age, Brinkman index, proportion of former current smokers, and pulmonary functions were not significantly different among 3 groups relative to ACTDA value (Tables 2 and 5). In male subjects aged ≥ 65, mean ages and proportion of former or current smokers were not significantly different among 3 groups (Table 3). Higher level of ACTDA was associated with higher Brinkman index, and lower % predicted FVC and % predicted FEV_1_ (Table 3). In female subjects ≥ 65 years of age, these differences were not observed (Table 6).

**Table 1:** Comparison of characteristics according to serum level of antibodies to several connective tissue disease autoantigens (ACTDA) in male subjects.

	**ACTDA < 20 (n = 1306)**	**ACTDA ≥ 20, < 40 (n = 173)**	**ACTDA ≥ 40 (n = 18)**	***P***
**Age, years**	62.4 (10.4)	65.1 (10.2)*	67.8 (10.7)	0.0007
**BI, cigarettes × years**	432.2 (489.6)	481.0 (462.8)	655.9 (932.3)	0.11
**Past/current smoker, %**	66	70.5	72.2	0.42
**FVC% predicted**	97.5 (14.7)	96.9 (15.9)**^*#*^**	87.7 (16.6)*	0.02
**FEV_1_% predicted**	95.9 (17.4)	94.7 (16.8)**^*#*^**	81.2 (22.2)*	0.002
**FEV_1_/FVC, %**	77.1 (8.9)	76.6 (8.6) **^*#*^**	71.3 (11.5) *	0.02
**MMF% predicted**	92.1 (37.6)	89.0 (34.6)	68.6 (32.0)*	0.02

Of 3,257 subjects who underwent spirometry, ACTDA values were not available for 10 subjects and Brinkman index was not available for 335 subjects because of the lack of precise information about cigarette smoking.

This table shows characteristics of all male subjects according to serum levels of ACTDA.

Values are means (SD) or percentage.

*, *P* < 0.05 vs ACTDA < 20; #, *P* < 0.05 vs ACTDA ≥ 40

Abbreviations: BI, Brinkman index; FVC, forced vital capacity; FEV_1_, forced expiratory volume in 1 s; MMF, maximal midexpiratory flow

**Table 2:** Comparison of characteristics according to serum level of antibodies to several connective tissue disease autoantigens (ACTDA) in male subjects aged younger than 65 years.

**Males** (**age <65**)	**ACTDA < 20 (n = 702)**	**ACTDA ≥ 20, < 40 (n = 71)**	**ACTDA ≥ 40 (n = 5)**	***P***
**Age, years**	54.5 (6.6)	54.9 (6.3)	54.4 (9.4)	0.89
**BI, cigarettes × years**	443.7 (440.4)	466.7 (452.4)	327.0 (338.2)	0.8
**Past/current smoker, %**	68.1	70.4	60	0.85
**FVC% predicted**	97.9 (12.5)	97.0 (12.7)	96.8 (11.6)	0.83
**FEV_1_% predicted**	97.2 (14.7)	96.5 (12.9)	93.4 (15.8)	0.78
**FEV_1_/FVC, %**	78.7 (7.4)	79.1 (7.3)	76.2 (4.8)	0.68
**MMF% predicted**	96.2 (33.2)	95.5 (31.9)	80.5 (25.9)	0.56

Of 3,257 subjects who underwent spirometry, ACTDA values were not available for 10 subjects and Brinkman index was not available for 335 subjects because of the lack of precise information about cigarette smoking.

This table shows characteristics of male subjects aged younger than 65 years according to serum levels of ACTDA.

Values are means (SD) or percentage.

Abbreviations: BI, Brinkman index; FVC, forced vital capacity; FEV_1_, forced expiratory volume in 1 s; MMF, maximal midexpiratory flow

**Table 3:** Comparison of characteristics according to serum level of antibodies to several connective tissue disease autoantigens (ACTDA) in male subjects aged 65 years and older.

	**ACTDA < 20 (n = 604)**	**ACTDA ≥ 20, < 40 (n = 102)**	**ACTDA ≥ 40 (n = 13)**	***P***
**Age, years**	71.6 (5.0)	72.3 (4.9)	73.0 (5.2)	0.34
**BI, cigarettes × years**	420.9 (512.2)	491.1 (472.6)	792.9 (1073.9)*	0.03
**Past/current smoker, %**	63.4	70.6	76.9	0.24
**FVC% predicted**	97.1 (16.8)	96.8 (17.9)**^*#*^**	84.1 (17.3)*	0.03
**FEV_1_% predicted**	94.3 (20.1)	93.5 (19.1)**^*#*^**	76.5 (22.9)*	0.006
**FEV_1_/FVC, %**	75.2 (10.0)	74.8 (9.1)	69.4 (12.9)	0.11
**MMF% predicted**	87.3 (41.5)	84.5 (35.8)	64.1 (33.8)	0.11

Of 3,257 subjects who underwent spirometry, ACTDA values were not available for 10 subjects and Brinkman index was not available for 335 subjects because of the lack of precise information about cigarette smoking.

This table shows characteristics of male subjects aged 65 years and older according to serum levels of ACTDA.

Values are means (SD) or percentage.

*, *P* < 0.05 vs ACTDA < 20; #, *P* < 0.05 vs ACTDA ≥ 40

Abbreviations: BI, Brinkman index; FVC, forced vital capacity; FEV_1_, forced expiratory volume in 1 s; MMF, maximal midexpiratory flow

**Table 4:** Comparison of characteristics according to serum level of antibodies to several connective tissue disease autoantigens (ACTDA) in female subjects.

	**ACTDA < 20 (n = 1374)**	**ACTDA ≥ 20, < 40 (n = 313)**	**ACTDA ≥ 40 (n = 59)**	***P***
**Age, years**	61.4 (10.3)	62.7 (10.1)	65.4 (8.5) *	0.003
**BI, cigarettes × years**	18.1 (91.9)	14.9 (84.9)	11.6 (62.5)	0.75
**Past/current smoker, %**	9.5	9	5.1	0.51
**FVC% predicted**	100.1 (14.2)	99.1 (14.3)	97.4 (17.4)	0.21
**FEV_1_% predicted**	100.0 (15.2)	99.1 (15.8)	97.0 (18.2)	0.24
**FEV_1_/FVC, %**	80.0 (6.4)	79.8 (6.7)	79.4 (8.0)	0.73
**MMF% predicted**	100.0 (32.8)	99.9 (37.1)	95.3 (35.5)	0.58

Of 3,257 subjects who underwent spirometry, ACTDA values were not available for 10 subjects and Brinkman index was not available for 335 subjects because of the lack of precise information about cigarette smoking.

This table shows characteristics of all female subjects according to serum levels of ACTDA.

Values are means (SD) or percentage.

* , *P* < 0.05 vs ACTDA < 20; #, *P* < 0.05 vs ACTDA ≥ 40

Abbreviations: BI, Brinkman index; FVC, forced vital capacity; FEV_1_, forced expiratory volume in 1 s; MMF, maximal midexpiratory flow

**Table 5:** Comparison of characteristics according to serum level of antibodies to several connective tissue disease autoantigens (ACTDA) in female subjects aged younger than 65 years.

	**ACTDA < 20 (n = 800)**	**ACTDA ≥ 20, < 40 (n = 166)**	**ACTDA ≥ 40 (n = 26)**	***P***
**Age, years**	54.2 (6.6)	54.6 (6.8)	57.3 (4.1)	0.05
**BI, cigarettes × years**	25.5 (103.1)	21.2 (93.8)	26.0 (92.3)	0.89
**Past/current smoker, %**	13.4	14.5	7.7	0.64
**FVC% predicted**	100.7 (13.1)	98.4 (13.0)	99.4 (13.9)	0.13
**FEV_1_% predicted**	100.5 (13.9)	97.9 (15.2)	97.9 (17.5)	0.09
**FEV_1_/FVC, %**	81.0 (5.6)	80.5 (6.4)	79.5 (8.1)	0.31
**MMF% predicted**	100.4 (29.4)	97.2 (32.5)	95.2 (31.0)	0.34

Of 3,257 subjects who underwent spirometry, ACTDA values were not available for 10 subjects and Brinkman index was not available for 335 subjects because of the lack of precise information about cigarette smoking.

This table shows characteristics of female subjects aged younger than 65 years according to serum levels of ACTDA.

Values are means (SD) or percentage.

Abbreviations: BI, Brinkman index; FVC, forced vital capacity; FEV_1_, forced expiratory volume in 1 s; MMF, maximal midexpiratory flow

**Table 6:** Comparison of characteristics according to serum level of antibodies to several connective tissue disease autoantigens (ACTDA) in female subjects aged 65 years and older.

	**ACTDA < 20 (n = 574)**	**ACTDA ≥ 20, < 40 (n = 147)**	**ACTDA ≥ 40 (n = 33)**	***P***
**Age, years**	71.4 (4.6)	71.8 (4.6)	71.7 (4.9)	0.59
**BI, cigarettes × years**	8.2 (73.3)	8.3 (74.1)	0.0 (0.0)	0.81
**Past/current smoker, %**	4	2.7	3	0.74
**FVC% predicted**	99.4 (15.6)	99.9 (15.6)	95.8 (19.8)	0.38
**FEV_1_% predicted**	99.3 (16.9)	100.3 (16.4)	96.2 (18.9)	0.45
**FEV_1_/FVC, %**	78.7 (7.2)	79.0 (6.9)	79.4 (8.1)	0.76
**MMF% predicted**	99.4 (37.0)	103.0 (41.6)	95.5 (39.2)	0.48

Of 3,257 subjects who underwent spirometry, ACTDA values were not available for 10 subjects and Brinkman index was not available for 335 subjects because of the lack of precise information about cigarette smoking.

This table shows characteristics of female subjects aged 65 years and older according to serum levels of ACTDA.

Values are means (SD) or percentage.

Abbreviations: BI, Brinkman index; FVC, forced vital capacity; FEV_1_, forced expiratory volume in 1 s; MMF, maximal midexpiratory flow

### Relationships between ACTDA and spirometric measures

**Figure 1:**
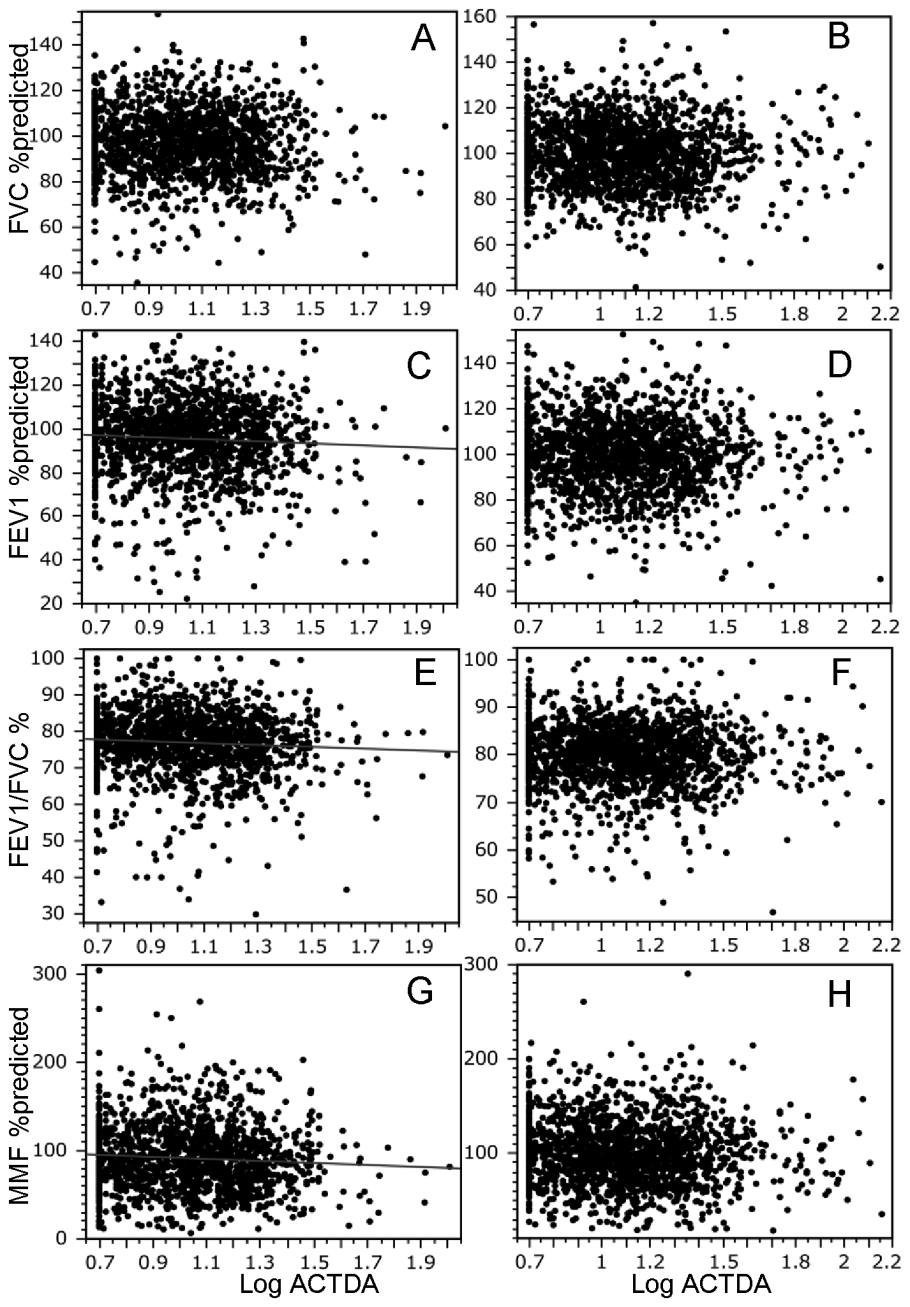
Correlations between logarithmically-transformed value of antibodies to several connective tissue disease autoantigens (ACTDA) and spirometric measures in all subjects in the study population. Graphs show the relationships between annual decline in spirometric parameters (A and B, % predicted FVC; C and D, % predicted FEV_1_; E and F, FEV_1_/FVC; G and H, % predicted MMF) and serum ACTDA value relative to sex (male: A, C, E, and G; female: B, D, F and H). A significant relationship of log ACTDA value was observed with FEV_1_, FEV_1_/FVC, and MMF in male subjects (A, b = -2.51, *P* = 0.124, β = -0.04; B, b = -1.88, *P* = 0.134, β = -0.04; C, b = -4.71, *P* = 0.014, β = -0.06; D, b = -1.86, *P* = 0.168, β = -0.03; E, b = -2.52, *P* = 0.001, β = -0.07; F, b= -0.42, *P* = 0.457, β = -0.02; G, b = -11.49, *P* = 0.005, β = -0.07 and H, b= -3.63, *P* = 0.218, β = -0.03). ACTDA values were not available for 10 subjects. n = 1498 and 1746 male and female subjects, respectively. b, partial regression coefficient; β, standard partial regression coefficient; FVC, forced vital capacity; FEV_1_, forced expiratory volume in 1 s; MMF, maximal midexpiratory flow.

We investigated the relationship between log ACTDA value and spirometric measures (Figure 1). As shown in Figure 1, there were significant correlations with FEV_1_% predicted, FEV_1_/FVC and MMF% predicted in males (Figure 1C, Figure 1E, and Figure 1G) but not with FVC% predicted in males or with FVC% predicted, FEV_1_% predicted, FEV_1_/FVC and MMF% predicted in females (Figure 1A, Figure 1B, Figure 1D, Figure 1F, and Figure 1H). The relationships of log ACTDA to pulmonary functions in individuals with ACTDA ≥ 20 and ≥ 40 have been summarized in Tables 7 and 8, respectively. The correlation of ACTDA ≥ 20 with % predicted FEV_1_ and % predicted MMF was significant in men but not in women (Table 7). However, the correlation of ACTDA ≥ 40 with spirometric parameters was not significant in men or women, probably because the number of individuals with ACTDA ≥ 40 was not large enough to demonstrate relationships between ACTDA and spirometric values (Table 8). Next, we evaluated whether these correlations were still significant after adjustment for Brinkman index using multiple linear regression analysis. This analysis revealed that log ACTDA value was significantly associated with % predicted FEV_1_ and % predicted MMF, independently of Brinkman index, but not with % predicted FVC and FEV_1_/FVC (Table 9). Multiple linear analyses were performed among individuals with ACTDA ≥ 20 and ≥ 40; however, probably because of insufficient numbers of these individuals, significant relationships between log ACTDA and pulmonary functions were not observed (data not shown).

**Table 7:** Relationships of logarithmically-transformed values of antibodies to several connective tissue disease autoantigens (ACTDA) to pulmonary functions in ACTDA-positive individuals: univariate linear regression analyses (ACTD ≥ 20).

	Men (n = 191)				Women (n = 372)			
	b	SE	*P*	β	b	SE	*P*	β
FVC% predicted	-15.51	9.3	0.097	-0.12	-0.53	4.43	0.905	0.01
FEV_1_% predicted	-23.73	10.15	0.021	-0.17	-0.49	4.84	0.919	-0.01
FEV_1_/FVC	-10.08	5.19	0.053	-0.14	-1.2	2.07	0.563	-0.03
MMF% predicted	-41.3	19.91	0.04	-0.15	-10.08	11.02	0.361	-0.05

Univariate linear regression analyses in ACTDA-positive individuals; explanatory variable, log ACTDA value; dependent variable, each spirometric parameter

Abbreviations: b, partial regression coefficient; β, standard partial regression coefficient; FVC, forced vital capacity; FEV_1_, forced expiratory volume in 1 s; MMF, maximal midexpiratory flow

**Table 8:** Relationships of logarithmically-transformed values of antibodies to several connective tissue disease autoantigens (ACTDA) to pulmonary functions in ACTDA-positive individuals: univariate linear regression analyses (ACTD ≥ 40).

	Men (n = 18)				Women (n = 59)			
	b	SE	*P*	β	b	SE	*P*	β
FVC% predicted	9.03	35.3	0.801	0.06	11.2	16.13	0.491	0.09
FEV_1_% predicted	20.4	46.83	0.669	0.11	2.76	16.94	0.871	0.02
FEV_1_/FVC	11.9	24.36	0.632	0.12	-6.74	7.43	0.368	-0.1
MMF% predicted	-6.4	67.99	0.926	-0.02	-29	32.89	0.382	-0.1

Univariate linear regression analyses in ACTDA-positive individuals; explanatory variable, log ACTDA value; dependent variable, each spirometric parameter

Abbreviations: b, partial regression coefficient; β, standard partial regression coefficient; FVC, forced vital capacity; FEV_1_, forced expiratory volume in 1 s; MMF, maximal midexpiratory flow

**Table 9:** Multiple linear regression analyses for the relationships between Brinkman index, antibodies to several connective tissue disease autoantigens (ACTDA) value, and spirometric measures in this study population.

**Explanatory v. (dependent v.)**	**b**	**SE**	***P***	**β**
BI (FVC% predicted)	-0.005	0.001	<0.0001	-0.14
Log ACTDA (FVC% predicted)	-1.8	1.028	0.08	-0.03
BI (FEV_1_% predicted)	-0.009	0.001	<0.0001	-0.22
Log ACTDA (FEV_1_% predicted)	-2.56	1.143	0.025	-0.04
BI (FEV_1_/FVC)	-0.004	0	<0.0001	-0.2
Log ACTDA (FEV_1_/FVC)	-0.413	0.523	0.43	-0.01
BI (MMF% predicted)	-0.019	0.002	<0.0001	-0.2
Log ACTDA (MMF% predicted)	-5.796	2.473	0.019	-0.04

Multivariate linear regression analyses in all study subjects; explanatory variable (v.), BI and log ACTDA value; dependent v., each spirometric parameter

FVC% predicted, FEV_1_% predicted and MMF% predicted were standardized values by age, sex, and height (Reference #10). FEV_1_/FVC was adjusted for age and sex.

Abbreviations: b, partial regression coefficient; β, standard partial regression coefficient; BI, Brinkman index; FVC, forced vital capacity; FEV_1_, forced expiratory volume in 1 s; MMF, maximal midexpiratory flow

### Comparison of ACTDA positivity among the spirometric classifications

To examine if positive ACTDA was the cause of restrictive or obstructive pulmonary disease in never-smokers, we compared ACTDA positivity among the following spirometric classifications: normal, % predicted FVC ≥ 80 and FEV_1_/FVC ≥ 0.7; restrictive, % predicted FVC < 80 and FEV_1_/FVC ≥ 0.7; obstructive, % predicted FVC ≥ 80 and FEV_1_/FVC <0.7; and mixed disorder, % predicted FVC < 80 and FEV_1_/FVC < 0.7. As shown in Table 10, the prevalence of ACTDA positivity was significantly different among the spirometric classifications only in male never-smokers. The prevalence of ACTDA positivity in pure restrictive and pure obstructive subjects was not significantly different from that of normal subjects. In contrast, the prevalence of ACTDA positivity was significantly greater in subjects with mixed pulmonary disorder than in other groups of male never-smokers. The mean age of subjects with mixed pulmonary disorder was significantly greater than that of normal subjects in male never-smokers. Among patients with COPD, residual volume and functional residual capacity increased exponentially as FEV_1_ decreased, while vital capacity and inspiratory capacity decreased linearly, suggesting that patients with obstructive-pattern COPD advance to mixed-pattern COPD with disease progression because of increased air trapping [22]. Thus, subjects with mixed disorder were considered to have more advanced airway obstruction than subjects with pure obstructive disorder in this study population. We therefore divided the subjects with airflow obstruction (FEV_1_/FVC < 0.7) into 2 groups by the median value of % predicted FEV_1_, and we compared the difference in prevalence of ACTDA positivity among normal subjects, subjects with less severe airway obstruction (% predicted FEV_1_ ≥ median value), and subjects with more severe airway obstruction (% predicted FEV_1_ < median value) (Table 11). The prevalence of ACTDA positivity was significantly greater only in male never-smokers with obstructive disorder and FEV_1_ below the median value, not in female never-smokers. The mean age of male never-smokers with obstructive disorder and FEV_1_ below the median value was significantly greater than that of normal subjects in male never-smokers.

**Table 10:** Prevalence of antibodies to several connective tissue disease autoantigens (ACTDA) positivity and mean age relative to the type of spirometric disorder, sex, and smoking status.

	**Total subjects number**	**Number of ACTDA ≥20**	**Prevalence of ACTDA ≥20 (%)**	**95% CI (%)**	**Age**
**Normal, male never-smokers**	439	45	10.3	7.7–13.4	63.3 (10.1)
**Restrictive, male never-smokers**	21	21	14.3	5.0–34.6	65.4 (11.9)
**Obstructive, male never-smokers**	31	3	9.7	3.3–24.9	67.7 (8.9)
**Mixed, male never-smokers**	10	5	50*	23.7–76.3	74.9 (5.3)*
**Normal, male smokers**	725	93	12.8	10.6–15.5	60.4 (10.5)
**Restrictive, male smokers**	75	10	13.3	7.5–23.1	64.9 (10.3)*
**Obstructive, male smokers**	153	24	15.7	7.5–23.1	67.6 (8.4)*
**Mixed, male smokers**	45	8	17.8	9.3–31.3	69.3 (6.5)*
**Normal, female never-smokers**	1415	296	20.9	18.9–23.1	62.0 (10.1)
**Restrictive, female never-smokers**	86	25	29.1	20.5–39.4	65.8 (10.2)*
**Obstructive, female never-smokers**	70	15	21.4	13.4–32.3	66.7 (9.8)
**Mixed, female never-smokers**	15	5	33.3	15.2–58.3	66.7 (7.5)
**Normal, female smokers**	135	28	20.7	14.8–28.3	53.7 (8.9)
**Restrictive, female smokers**	13	1	7.7	1.3–33.3	57.3 (12.2)
**Obstructive, female smokers**	10	1	10	1.8–40.4	63.6 (10.2)*
**Mixed, female smokers**	3	1	33.3	6.1–79.2	60.7 (7.8)

Age: mean years-old (standard deviation)

Smokers included both past and current smokers.

ACTDA values were not available in 2 male never-smokers, 1 male past/current smoker, and 7 female never-smokers.

Differences in the percentage of positive ACTDA and the age by type of spirometric disorder were evaluated using the chi-square test and analysis of variance followed by Tukey’s test, respectively. *: *P* < 0.05 vs “Normal” group.

Abbreviations: CI, confidence interval

**Table 11:** Prevalence of antibodies to several connective tissue disease autoantigens (ACTDA) positivity among never-smokers with normal spirometry findings, those with obstructive spirometry findings equal or above median of % predicted FEV_1_, and those below the median value.

	**Total subjects number**	**Number of ACTDA ≥20**	**Prevalence of ACTDA ≥20**	**95% CI (%)**	**Age**
**Normal, male never-smokers**	439	45	10.3	7.7–13.4	63.3 (10.1)
**Obstructive and %FEV_1_ ≥ median, male never-smokers**	22	2	9.1	2.5–27.8	67.3 (9.1)
**Obstructive and %FEV_1_ < median, male never-smokers**	19	6	31.6*	15.4–54.0	71.9 (7.8)*
**Normal, female never-smokers**	1415	296	20.9	18.9–23.1	62.0 (10.1)
**Obstructive and %FEV_1_ ≥ median, female never-smokers**	45	11	24.4	14.2–38.7	66.3 (10.0)*
**Obstructive and %FEV_1_ < median, female never-smokers**	40	9	22.5	12.3–37.5	67.1 (8.7)*

Age: mean years-old (standard deviation)

Median values of % predicted FEV_1_ among subjects with obstructive disorder were 75.35% in males and 77.15% in females.

ACTDA values were not available in 2 male never-smokers, 1 male past/current smoker, and 7 female never-smokers.

Differences in the percentage of positive ACTDA and the age by type of spirometric disorder were evaluated using the chi-square test and analysis of variance followed by Tukey’s test, respectively. *: *P* < 0.05 vs “Normal” group.

Abbreviations: CI, confidence interval; FEV_1_, forced expiratory volume in 1 s

### Relationship between ACTDA and decline in spirometric values

The relationship between decline in spirometric values and the logarithmically transformed value of ACTDA was investigated. As shown in Table 12, a higher ACTDA value was associated with greater loss of % predicted FVC and % predicted FEV_1_. After adjustment for Brinkman index, statistically significant differences disappeared, although there were similar trends without statistical significance (Table 13).

**Table 12:** Univariate linear regression analyses: relationships between decline in spirometric values and logarithmically-transformed value of antibodies to several connective tissue disease autoantigens (ACTDA).

	b	SE	*P*	β
Change in FVC (%/year)	-2.061	0.962	0.034	-0.18
Change in FEV_1_ (%/year)	-2.283	1.146	0.048	-0.16
Change in FEV_1_/FVC (%/year)	-0.167	0.774	0.829	-0.02
Change in MMF (%/year)	-3.816	3.383	0.261	-0.09

Relationships between spirometric measures and ACTDA values were analyzed using univariate linear regression analyses. Explanatory variable, log ACTDA value; dependent variable, each decline in spirometric parameter

n = 147

Abbreviations: b, partial regression coefficient; β, standard partial regression coefficient; FVC, forced vital capacity; FEV_1_, forced expiratory volume in 1 s; MMF, maximal midexpiratory flowβ, standard partial regression coefficient

**Table 13:** Multivariate linear regression analyses: relationships between decline in spirometric values and logarithmically-transformed value of antibodies to several connective tissue disease autoantigens (ACTDA).

	b	SE	*P*	β
Change in FVC (%/year)	-1.715	1.037	0.101	-0.15
Change in FEV_1_ (%/year)	-1.688	1.015	0.099	-0.15
Change in FEV_1_/FVC (%/year)	-0.036	0.763	0.962	-0.01
Change in MMF (%/year)	-0.993	2.402	0.679	-0.04

Relationships between spirometric measures and ACTDA values were analyzed using multivariate linear regression analyses. Explanatory variable, log ACTDA value and Brinkman index; dependent variable, each decline in spirometric parameter

n = 147

Abbreviations: b, partial regression coefficient; β, standard partial regression coefficient; FVC, forced vital capacity; FEV_1_, forced expiratory volume in 1 s; MMF, maximal midexpiratory flowβ, standard partial regression coefficient

## Discussion

In the present study, we demonstrated an inverse relationship between spirometric measures and ACTDA value in male subjects participating in an annual health check-up. The relationships between ACTDA value and FEV_1_, and MMF remained significant after adjustment for Brinkman index. In male never-smokers, the prevalence of ACTDA positivity was significantly greater in subjects with more severe airway obstruction (% predicted FEV_1_ < the median value) than in normal subjects and subjects with less severe airway obstruction (% predicted FEV_1_ ≥ the median value), although it is possible that the difference in percentage of male never-smokers positive for ACTDA may be attributable to the difference in the mean age of each group.

Accumulating evidence has suggested the involvement of autoimmunity in the pathogenesis of COPD. In addition, some autoimmune diseases cause restrictive lung disorders, namely, interstitial pneumonias [23]. However, to date, the relationship between pulmonary function and autoimmunity has not been shown in a healthy population. This report is the first to demonstrate this significant relationship, which suggests the involvement of an autoimmune mechanism in the impairment of pulmonary function, especially in obstructive lung disorders. As shown in Table 9, standard partial regression coefficient values of log ACTDA were smaller than those of the Brinkman index, but significant in relation to FEV1 and MMF. In addition, the positivity of ACTDA was high in the male never-smokers with severe obstruction (Table 11). Therefore, in some subjects with airflow obstruction, autoimmunity may be involved in its pathogenesis.

The precise causes and diagnoses of the pulmonary dysfunction/restrictive lung disorders were not clear in the present study because this study was not hospital-based research and such information was not available. There may be various causes of restrictive pulmonary disorders in Japan, and the prevalence of interstitial pneumonias is low [24]. Some portion of this population may have contracted interstitial pneumonias due to autoimmune diseases. However, there were no significant relationship between log ACTDA and FVC% predicted in the univariate linear regression analysis (Figure 1, Tables 7 and 8) and multivariate linear regression analysis (Table 9). In addition, the prevalence of positive ACTDA in restrictive lung disorder was similar to that in normal subjects (Table 10). Thus, we could not confirm a significant relationship between ACTDA level and FVC, the spirometric parameter for restrictive lung disorder, as we could for the relationship between ACTDA and FEV_1_, the spirometric parameter for obstructive lung disorder. 

It is reasonable to assume that inhalation of cigarette smoke induces impairment of pulmonary function. In this study, we demonstrated a significant positive relationship between ACTDA value and Brinkman index in healthy male subjects, although the habit of smoking did not related to the level of ACTDA (Tables 1 and 4) or its positivity (data not shown). In addition, inverse associations between logarithmically transformed ACTDA value and % predicted FEV_1_ and MMF were demonstrated independently of Brinkman index. These results suggest that cigarette smoking does not initiate ACTDA positivity but enhances serum ACTDA levels, and that this elevation of ACTDA plays a significant role in the impairment of pulmonary function in male smokers independent of the direct toxic effect of cigarette smoke on pulmonary cells. In females, these correlations were not observed. A lower rate of cigarette smoking and lower Brinkman index in women than men may be a part of the reason for this difference between the sexes.

A primary cause of obstructive disorders may be COPD or bronchial asthma. In Japan, the prevalence of COPD and bronchial asthma is estimated from 8% to 10% and from 1% to 3% in adults, respectively [25,26]. Bronchial asthma is a comorbidity in allergic granulomatous angiitis (AGA), in which elevation of anti-neutrophil cytoplasmic antibody is seen, although there are no clear links between bronchial asthma and other autoimmune diseases. In addition, the prevalence of AGA is very low [27]. However, the prevalence of positive ANA is higher among asthmatic patients than among healthy subjects, suggesting that the involvement of an autoimmune mechanism in some asthmatic patients [28]. COPD is recognized as a chronic inflammatory disease [29]. The pathogenesis of COPD has traditionally been attributed to the involvement of alveolar macrophages, neutrophils, and CD8 T cells [30]. Recently, the involvement of adaptive immunity has been suggested by several investigators [4,31]. In particular, the presence of ANA and organ-specific antibodies in COPD patients has also been shown by several investigators [5,7,32-34].

In contrast to the positive relationship between ACTDA value and Brinkman index in the present study, we demonstrated that the prevalence of ACTDA positivity was greater in male never-smokers with relatively severe airflow obstruction. Of 501 male never-smokers, 41 subjects had airflow obstruction (31, obstructive; 10 mixed), and 8 of those subjects were positive for ACTDA. The prevalence of ACTDA positivity was greater in male never-smokers with the mixed pulmonary disorder than in subjects with other spirometric patterns. Autoimmunity has been suggested to be involved in the progression of COPD after cessation of cigarette smoking [6]. However, to date, there is little evidence supporting the involvement of autoantibodies in non-smoking COPD patients [7]. In this study, the prevalence of positive ACTDA was greater in male never-smokers with more severe airflow obstruction. This suggests the involvement of autoimmunity, not only in the pathogenesis of smoking-related pulmonary dysfunction but also in that of obstructive pulmonary diseases in male non-smokers. Subjects with mixed disorder and subjects with more severe airflow obstruction were older than subjects in other groups. Higher prevalence of positive ACTDA in the subjects in these groups is merely due to the higher mean ages of these groups. However, subjects in the greater ACTDA group had lower % predicted FVC and FEV_1_ in males aged 65 and older, while the mean ages did not differ among groups that were determined by the level of ACTDA (Table 3). Log ACTDA was significantly associated with % predicted FEV_1_ and MMF, even if age and sex were included as covariates in the multiple linear regression analysis shown in Table 9 (data not shown), suggesting that ACTDA is a factor associated with FEV_1_ and MMF that is independent of age. Thus, although we could not eliminate the possibility that age difference affected the positivity of ACTDA in Tables 10 and 11, the association between greater ACTDA positivity and respiratory dysfunction in male never-smokers is still thought to be possible. The progression of pulmonary impairment induced by autoimmunity in male never-smokers may be slow, and it may take very long time to become apparent as spirometric dysfunction. In female never-smokers, a significantly high prevalence of positive ACTDA was not observed in the subjects with pulmonary dysfunction. The precise reasons for this difference between males and females are unknown, but higher baseline levels of ACTDA in female subjects may hide the relationships between ACTDA levels and respiratory functions.

Severity of airflow obstruction is usually evaluated by GOLD classification [1], and when we compared the difference in ACTDA positivity using GOLD classification, such as GOLD1 (% predicted FEV_1_ ≥ 80) vs. GOLD2–4 (% predicted FEV_1_ < 80), this statistical significance was diminished (P = 0.06). However, there appears to be no clear scientific support for setting the boundary between GOLD1 (mild airflow limitation) and GOLD2 (moderate airflow limitation) at a % predicted FEV_1_ of 80% because there is no prognostic difference between them [35]. In addition, GOLD classification does not adequately reflect the individual severity of COPD with respect to dyspnea, health-related quality of life, exercise capacity, and frequency of exacerbation [36]. Therefore, we did not find the GOLD classification to be the optimal way to classify the population in the present study.

As mentioned earlier, the fact that the present study was not hospital based presents several limitations, including the lack of information regarding patients’ (i) history of autoimmune disease; (ii) findings on chest radiography; (iii) current medications, including immunosuppressive agents such as corticosteroids; and (iv) socio-economic status, which has the potential to create sampling bias. Furthermore, we did not measure ANA levels by IIF using HEp-2 cells, which is used in clinical environments to confirm the presence of ANA. The MESACUP ANA ELISA test performed in this study uses a mixture of only 9 antigens recognized by autoantibodies in systemic rheumatic diseases. In contrast, antibodies to hundreds to thousands of antigens are detected in a standard immunofluorescence ANA screening test. Only a small subset of individuals positive for ACTDA by immunofluorescence have autoantibodies to some of the 9 antigens used in MESACUP ANA EIA. In the future, IIF measurement of ANA and disease-specific antibodies should be evaluated in subjects with airflow obstruction without a history of cigarette smoking.

Because this study was performed in a sample of the Japanese population, it is still unknown if this relationship between ACTDA and respiratory function would exist for other ethnicities. ANA levels are reported to be twice as high in Japan than in the USA. Genetic backgrounds including human leukocyte antigens; and lifestyles, such as diet and smoking habit, are different in each ethnicity. Therefore, further studies are needed to confirm this correlation between autoimmunity and respiratory functions for other ethnicities.

In the longitudinal analysis, although significant associations between decline in FVC and FEV1 and ACTDA were observed, these associations seem to be weak because statistical significance did not remain after adjustment for Brinkman Index. The number of subjects participating in the longitudinal study (n= 147) might not have been large enough to achieve statistical significance in this assay. In the future, studies using larger numbers of subjects may be needed to evaluate the relationships between longitudinal changes in spirometric values and autoimmunity.

In conclusion, we have demonstrated inverse relationships between ACTDA value and spirometric parameters in healthy male subjects participating in an annual health checkup, suggesting the involvement of an autoimmune mechanism in the development of pulmonary dysfunction.
